# The uncoupled ATPase activity of the ABC transporter BtuC_2_D_2_ leads to a hysteretic conformational change, conformational memory, and improved activity

**DOI:** 10.1038/srep21696

**Published:** 2016-02-22

**Authors:** Nurit Livnat-Levanon, Amy I. Gilson, Nir Ben-Tal, Oded Lewinson

**Affiliations:** 1 Rappaport Research Institute, Department of Biochemistry, The Bruce and Ruth Rappaport Faculty of Medicine, Technion-Israel Institute of Technology, Haifa, Israel; 2 Department of Chemistry and Chemical Biology, Harvard University, Cambridge, MA, USA; 3 Department of Biochemistry and Molecular Biology, Tel-Aviv University, Tel-Aviv, Israel

## Abstract

ABC transporters comprise a large and ubiquitous family of proteins. From bacteria to man they translocate solutes at the expense of ATP hydrolysis. Unlike other enzymes that use ATP as an energy source, ABC transporters are notorious for having high levels of basal ATPase activity: they hydrolyze ATP also in the absence of their substrate. It is unknown what are the effects of such prolonged and constant activity on the stability and function of ABC transporters or any other enzyme. Here we report that prolonged ATP hydrolysis is beneficial to the ABC transporter BtuC_2_D_2_. Using ATPase assays, surface plasmon resonance interaction experiments, and transport assays we observe that the constantly active transporter remains stable and functional for much longer than the idle one. Remarkably, during extended activity the transporter undergoes a slow conformational change (hysteresis) and gradually attains a hyperactive state in which it is more active than it was to begin with. This phenomenon is different from stabilization of enzymes by ligand binding: the hyperactive state is only reached through ATP hydrolysis, and not ATP binding. BtuC_2_D_2_ displays a strong conformational memory for this excited state, and takes hours to return to its basal state after catalysis terminates.

Under various physiological circumstances enzymes need to be active and remain fully functional for a long time. Such is the case of housekeeping enzymes that are known to be constantly active for several hours or even days^1^,^2^,^3^,^4^,^5^,^6^, or any enzyme with a long half-life that performs an essential cellular task.

An analogy is often drawn between man-made machines and enzymes: both consume energy, perform work, and have moving parts. Quite routinely we observe that the performance of man-made machines deteriorates over time, especially when used extendedly. Intuitively, one would predict that a molecular machine, an enzyme, would also be adversely affected by prolonged work. To our knowledge, this expectation was never experimentally tested. Here, we studied the effects of prolonged activity on the stability and function of one enzyme, an *E. coli* ABC transporter.

From bacteria to man, ATP Binding Cassette (ABC) transporters comprise a very large and diverse protein super family^7^,^8^,^9^,^10^,^11^. They are involved in many physiological processes such as signaling, cell division, antigen presentation, and solute translocation^12^,^13^,^14^. ABC transporters are also the focus of much clinical research, being directly implicated in human disease, resistance of tumors to chemotherapy, bacterial resistance to antibiotics, and bacterial pathogenesis and virulence^8^,^14^,^15^. ABC transporters generate substrate concentration gradients across biological membranes at the expense of ATP hydrolysis^16^,^17^. One would expect that the energy input (ATP hydrolysis) would be tightly coupled to the output of work (substrate translocation). However, with a handful of exceptions^18^,^19^, ABC transporters are notorious for having high levels of ATPase activity in the absence of their transportable substrate. Thus, the enzyme is active, constantly hydrolyzing ATP without generating a substrate concentration gradient. This uncoupled ATPase activity has been observed in mammalian, yeast, and bacterial systems, with exporters and importers, in membrane vesicles, liposomes, and lipid nanodiscs^16^,^17^,^20^,^21^,^22^,^23^. The origin of this high basal ATPase activity is unknown. One possibility is the presence of unidentified endogenous substrates (*e.g.,* lipids) that stimulate the ATPase activity of the transporters. This might be the case for the highly pleiotropic yeast exporter Pdr5 that recognizes hundreds of different compounds^24^,^25^. However, this possibility is unlikely in the case of a highly specific importer such as the vitamin B_12_ importer^26^. Here, we studied the effects of constant, uncoupled ATP hydrolysis on the structural and functional integrity of the *E. coli* vitamin B_12_ transporter BtuC_2_D_2_.

We let BtuC_2_D_2_ hydrolyze ATP for minutes, hours, or days and followed its activity levels.

The results are very surprising: constant and prolonged ATP hydrolysis drives a hysteretic conformational change that leads to a hyperactive state, in which BtuC_2_D_2_ is more active than it was to begin with. This hyperactive state can only be reached via ATP hydrolysis and not by ATP binding. Thus, the phenomenon we describe here is very different from stabilization of proteins by ligand binding.

## Results

### The effects of prolonged activity on BtuC_2_D_2_

To study the effects of prolonged activity on BtuC_2_D_2_ we purified the core transporter (ATPase+permease) and then dialyzed it in a micro-dialysis tube against a buffer containing detergent and a mixture of *E. coli* lipids and L-α-Phosphatidylcholine from egg yolk (see methods for details). The lipids are added to prevent protein delipidation and the detergent prevents formation of sealed liposomes thus circumventing accessibility issues. Initially, all incubations/conditioning of BtuC_2_D_2_ were performed in this lipid/detergent environment. In later experiments, conditioning of BtuC_2_D_2_ was also performed in the absence of detergent, using reconstituted protein-lipid nanodiscs (see below).

Since our initial goal was to test whether constant activity accelerates the deterioration rate of BtuC_2_D_2_ we first tested two simple conditioning regimes, termed “resting” and “working”. In the “working” regime, the protein is dialyzed against the lipid/detergent buffer that contains magnesium and ATP. ATP is present at near saturating concentration (100 μM, 90% saturation, Supplementary Fig. 1A), and similar results were obtained when 250 μM ATP was used (94% saturation). Due to the high basal activity of BtuC_2_D_2_ the protein constantly hydrolyzes ATP^16^,^17^. The large volume of the buffer (1000-fold excess), the molar excess of ATP over BtuC_2_D_2_ (12,000:1), and the buffer’s periodic replacement ensures a constant supply of fresh ATP. We also verified that during the time course of the experiment ATP is not depleted and that there is no measurable inhibition by the hydrolysis products (Supplementary Fig. 1B). The “resting” regime is identical, containing magnesium but omitting ATP. Therefore, the protein sits idle during the conditioning.

At intervals, we withdrew samples, and to remove possible aggregates, we centrifuged them first at medium speed (10 min, 17,000xg) and then at high-speed (30 min, 150,000xg). Using either centrifugation speed we found that hardly any protein was pelleted, indicating little to no aggregation, and therefore routinely used the lower speed spin. We also used size exclusion chromatography to assess the aggregation state of the rested and worked samples and found that both lacked significant aggregation (Supplementary Fig. 2A). We later found that the protein that had aggregated and precipitated during the incubations could not be removed simply by pipetting (see later).

**Figure 1:**
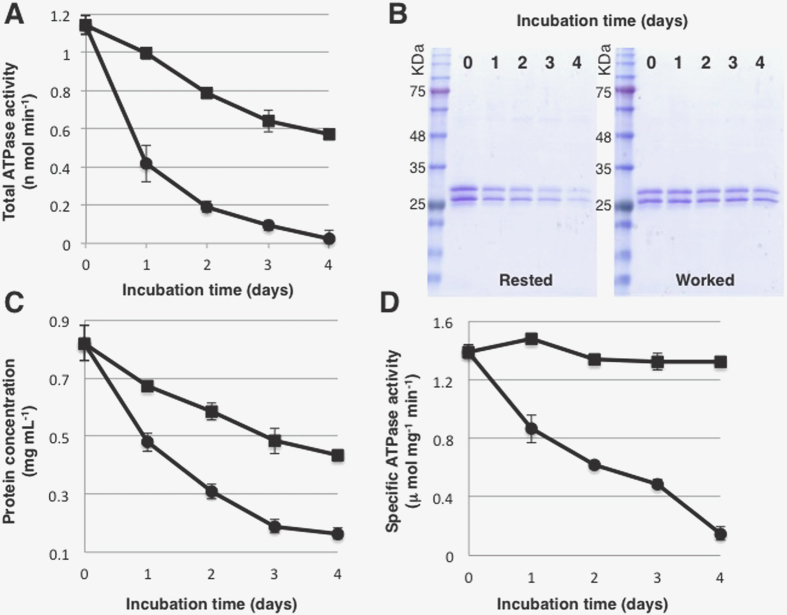
Activity and stability of the working and resting BtuC_2_D_2_. (**A**) BtuC_2_D_2_ was incubated at 28 °C under the resting (circles) and working (squares) regimes. At the indicated times equal VOLUMES were removed from each sample and assayed for hydrolysis of 1mM ATP. (**B**) Equal volumes from the samples used in A were subjected to reducing SDS-PAGE and stained by Coomassie blue. The two gels were identically run, stained, and imaged. (**C**) The protein concentration of the samples used in A was determined using a Lowry-based lipid and detergent compatible protein assay. Resting regime: circles, working regime: squares. (**D**) Specific ATPase activity under the resting (circles) and working (squares) regimes. Each data point shown in panel (**A**) was standardized by the remaining protein concentration shown in (**C**). The experiments were repeated at least three times and error bars represent standard deviations of technical triplicates of a single experiment.

We then measured how well BtuC_2_D_2_ that avoided aggregation retained its ATPase activity under the ‘resting’ or ‘working’ regimes. The results were very surprising: when we measured the total ATPase activity remaining in solution we found it to be much lower for the protein that had rested (Fig. 1A). Two factors contributed to the faster decline in total ATPase activity of the resting protein. First, less of the resting protein remained in solution over time. This was readily observed by Coomassie staining of SDS-PAGE (Fig. 1B) and by quantification of protein amounts using a detergent/lipid compatible protein assay (Fig. 1C). We did not observe protein degradation in either of the incubation regimes. Rather, we found that BtuC_2_D_2_ aggregates and precipitates during the incubations, and could only be extracted from the dialysis tubes with 10% SDS at 55 °C. In-line with its lower amounts of soluble protein, 10% SDS extracted more protein from the dialysis tube of resting BtuC_2_D_2_ (Supplementary Fig. 2B). A band of ~60 kDa appeared in these SDS-extracted samples. This band, which likely represents SDS-resistant higher molecular weight oligomers was much more prominent in the rested sample.

However, the decline in the amount of rested BtuC_2_D_2_ did not fully account the loss of activity: When we calculated the specific ATPase activity of the rested BtuC_2_D_2_ molecules we found that they gradually become inactive (Fig. 1D). Thus, with time, even those resting BtuC_2_D_2_ molecules that avoid precipitation and aggregation become inactive. This lower specific activity of rested BtuC_2_D_2_ is likely due to local unfolding events that compromise catalysis but do not yet lead to complete unfolding and aggregation.

In contrast, the working molecules that persisted in solution fully retained their specific ATPase activity throughout the 4 days of the experiment (Fig. 1D). Therefore, not only that the working molecules remain in solution longer (Fig. 1B, C), the specific activity of the persisting molecules is higher (Fig. 1D). The experiment shown in Fig.1 was conducted in 28 °C, yet we observed similar trends also at 20 and 37 °C, with the rates of activity loss accelerating with increasing temperatures (Supplementary Fig. 3). The working protein was also much superior to the idle one in the closely related functions of GTP and CTP hydrolysis (Supplementary Fig. 4). This is perhaps not surprising as hydrolysis of ATP, GTP, and CTP likely takes place at the same active site (the nucleotide binding domain of the transporter) and probably by a similar mechanism^27^.

**Figure 2:**
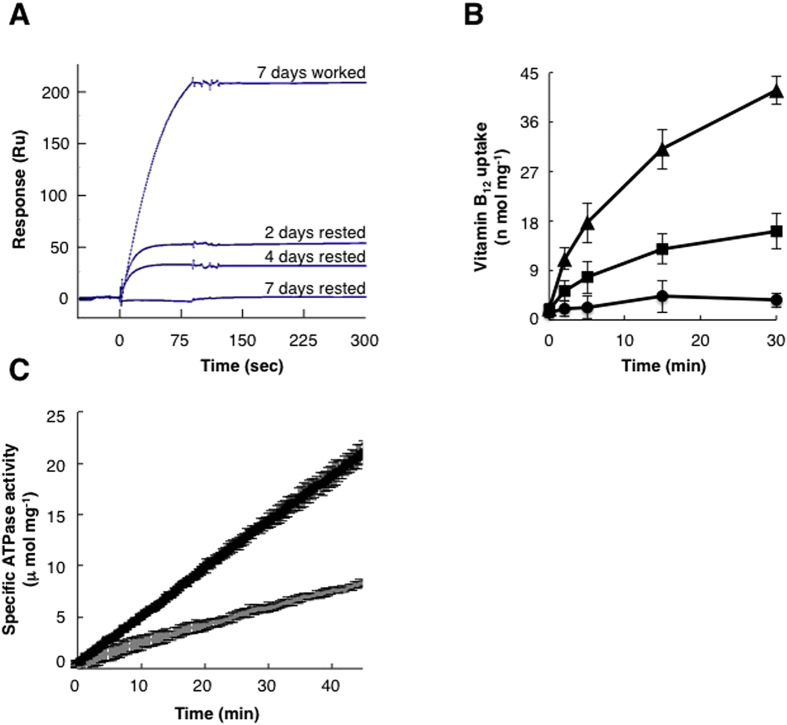
(**A**) Surface plasmon resonance analysis of the interaction between BtuF and BtuC_2_D_2_ that had rested or worked for the indicated times at 28 °C. At time zero, 0.2 μM apo BtuF is injected for 90 seconds over identical amounts of worked or rested BtuC_2_D_2_. (**B**) Vitamin B_12_ uptake by BtuC_2_D_2_ that had rested or worked. BtuC_2_D_2_ was incubated for 3 days at 28 °C at the working or resting conditions, and consequently equal protein amounts were reconstituted into liposomes. Uptake of 5 μM Co^57^ vitamin B_12_ (in the presence of 1 μM BtuF) by empty liposomes (circles), rested BtuC_2_D_2_ liposomes (squares), or worked BtuC_2_D_2_ liposomes (triangles) was measured using the rapid filtration method. Error bars represent standard deviations of three experiments. (**C**) ATPase activity of BtuC_2_D_2_ that was reconstituted into nanodiscs and then incubated for 3 days at 28 °C at the working (black trace) or resting (grey trace) conditions. The experiments were repeated at least three times and error bars represent standard deviations of technical triplicates from a single experiment.

We next tested rested and worked BtuC_2_D_2_ molecules for a function that occurs ~60 Å away from the site of ATP hydrolysis: binding of BtuF, the system’s substrate binding protein (SBP)^17^,^26^,^28^. Like all ABC importers BtuC_2_D_2_ must associate with its cognate SBP since it is the latter that binds the substrate and delivers it to the transporter. We measured the interaction between BtuC_2_D_2_ that had worked or rested at 28 °C and BtuF using Surface Plasmon Resonance^29^. We loaded identical amounts (by mass) of worked or rested BtuC_2_D_2_ onto a biosensor chip and then washed the chip 50,000-fold to remove any residual ATP. As shown (Fig. 2A), BtuC_2_D_2_ that had hydrolyzed ATP for 7 days at 28 °C robustly interacted with BtuF, while the 7-days rested protein was completely inactive. The 2-days and 4-days rested BtuC_2_D_2_ molecules interacted with BtuF but at reduced levels (Fig. 2A), in agreement with their reduced ATPase activities (Fig. 1D). Since identical amounts of non-aggregated rested or worked BtuC_2_D_2_ were loaded to the biosensor chip this shows that also with respect to BtuF binding the rested protein has a much-reduced specific activity.

We then measured the vitamin B_12_ transport activity of rested or worked BtuC_2_D_2_. For this we incubated BtuC_2_D_2_ for 3 days at 28 °C at the working or resting conditions, determined the amount of remaining protein, and then reconstituted equal protein amounts into liposomes. We also verified that equal amounts of rested or worked BtuC_2_D_2_ were indeed incorporated into the liposomes by washing the liposomes with sodium carbonate (to remove peripherally associated protein) and then subjecting them to sucrose flotation assays (to differentiate between aggregates and membrane-inserted protein). As shown in supplementary Fig. 5A,B the protein content of liposomes reconstituted with worked or rested BtuC_2_D_2_ was very similar. We incorporated an ATP-regenerating system in the lumen of the liposomes, and BtuF and^57^ Co-labeled vitamin B_12_ were added externally (see methods). BtuC_2_D_2_-mediated uptake of vitamin B_12_ was then measured using the rapid filtration method as others and we have performed in the past^16^,^17^. As shown, the transport activity of BtuC_2_D_2_ that had been incubated for 3 days under the working regime was 3–4 folds greater than that of the protein that had rested (Fig. 2B). As with the ATPase assays (Fig. 1D) and SPR experiments (Fig. 2A), this experiment shows that the specific activity of worked BtuC_2_D_2_ is higher than that of rested BtuC_2_D_2_.

In the experiments described above the conditioning/incubation of BtuC_2_D_2_ was conducted in a lipid/detergent solution. We next tested if a similar process occurs in a more native-like membrane environment. For this we reconstituted BtuC_2_D_2_ into lipid nanodiscs using previously established protocols^30^,^31^. Then, we subjected BtuC_2_D_2_-nanodiscs to the same working and resting regimes. Importantly, unlike the experiments described above, this incubation was performed in the absence of detergent. Fig. 2C shows that also in the absence of detergent, and in a membrane-mimetic environment the protein that had worked remained much more active than the one that had rested.

In summary, the data presented so far strongly demonstrates that prolonged activity does not accelerate the deterioration rate of BtuC_2_D_2_. We find this unexpected considering the constant conformational changes associated with ATP hydrolysis, flux of energy, and production of heat.

### Nucleotide binding stabilizes BtuC_2_D_2_

**Figure 3:**
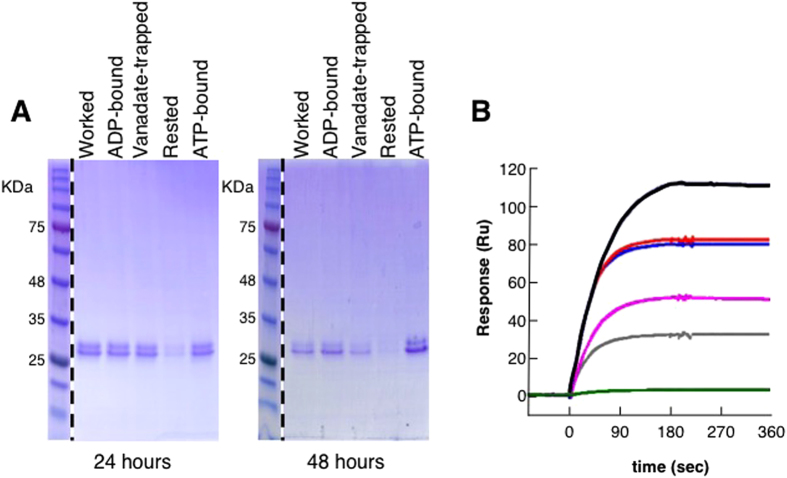
Nucleotide binding stabilizes BtuC_2_D_2_. (**A**) BtuC_2_D_2_ was incubated at the indicated conditions at 37 °C for 24 or 48 hours, as indicated. The amount of protein that remained in the soluble fraction visualized by Coomassie staining of reducing SDS-PAGE. The molecular weight marker is a cut and paste from the exact same gels. (**B**) BtuC_2_D_2_ was incubated for 1 day at 37 °C under the following regimes: resting (green), vanadate-trapped transition state (gray), ADP-bound (magenta), ATP-bound (red), or working (black). Also shown for comparison is the fresh (naïve) protein (blue). The nucleotides were removed by desalting and identical amounts (by mass) from each treatment were loaded onto an SPR biosensor chip. Following their immobilization the samples were washed ~50,000-fold to remove any residual ligands. At time zero 0.1 μM substrate-free BtuF was injected over all samples for 180 seconds. The experiments were repeated at least three times.

When hydrolyzing ATP BtuC_2_D_2_ repeatedly cycles through several nucleotide-bound conformations. Therefore, a likely explanation for the increased stability in the working conditions is that one (or several) of these conformations is more stable than the apo state. Thermodynamic stabilization of enzymes by their ligands is a very common phenomenon and was also observed in ABC transporters and other ATPases^32^. We therefore tested if one of the intermediate states of ATP hydrolysis (ATP-bound, transition state, or ADP-bound^33^) is responsible for the stabilization of ATP-hydrolyzing BtuC_2_D_2_. To test this, we conducted experiments where, in addition to the resting and working regimes, we included conditions that mimic the nucleotide-bound intermediates (see methods for details). Indeed, relative to the apo (‘resting’) state, in all of the nucleotide bound states, more protein persisted in solution over time (Fig. 3A). To measure the activity of the samples that had been pre-treated with nucleotide (worked, ATP-bound, transition state, or ADP-bound) we first removed the nucleotides with which they were incubated (see methods). We also verified by spectroscopy and gel filtration chromatography that the nucleotides had indeed been removed (Supplementary Fig. 6A), and that there is no aggregation (Supplementary Fig. 6B). Figure 3B shows that relative to the apo state (‘resting’ protein), all of the nucleotide bound states better preserve the BtuF binding activity of BtuC_2_D_2_. Taken together, these experiments show that the stabilization of BtuC_2_D_2_ under conditions of constant ATP hydrolysis is probably due to nucleotide binding.

### Only ATP hydrolysis, and not binding, leads to a hyperactive state

**Figure 4:**
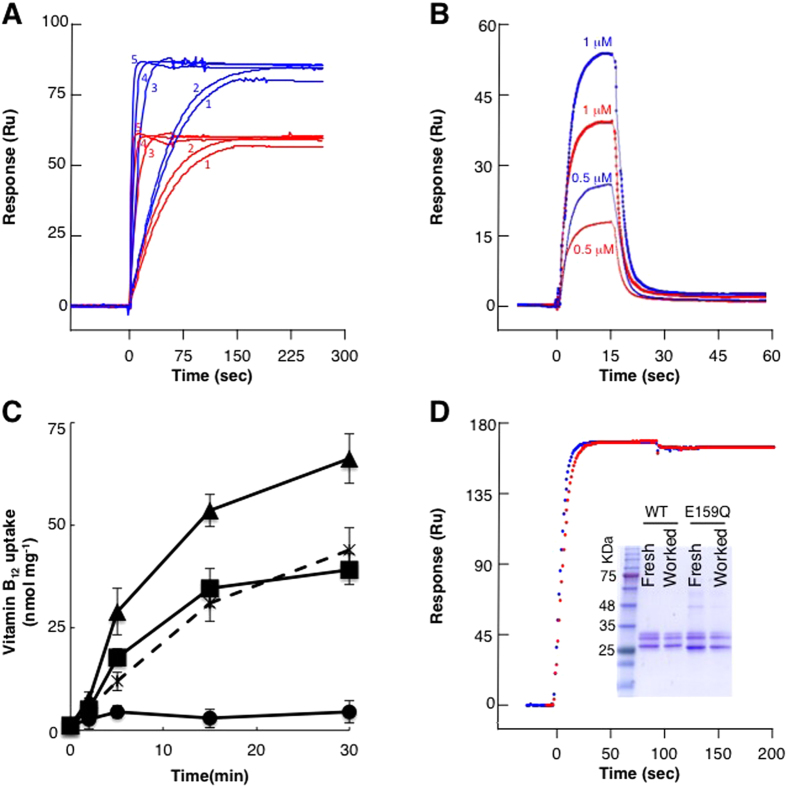
BtuC_2_D_2_ that has worked for several hours outperforms its naïve precursor. (**A**) Identical amounts of fresh BtuC_2_D_2_ (red curves) or the same preparation that has worked for 7 hours (blue curves) were loaded onto an SPR chip. At time zero a series of five apo BtuF concentrations was injected, as indicated next to each curve: (1) 0.1 μM, (2) 0.175 μM, (3) 0.25 μM, (4) 0.75 μM, (5) 2 μM (**B**) Same as in (**A**) only holo BtuF was injected at 0.5 μM or 1 μM (as indicated) in the presence of 200 μM vitamin B_12_. (**C**) Uptake of ^57^ Co vitamin B_12_ into empty liposomes (circles), liposomes reconstituted with fresh BtuC_2_D_2_ (squares), BtuC_2_D_2_ that was pre-incubated in the ATP-bound state for 7 hours (crosses), or with BtuC_2_D_2_ that has worked for 7 hours (triangles). (**D**) Identical amounts of fresh BtuC_2_D_2_ mutant E159Q (red curve) or the same preparation that was incubated for 7 hours at the ATP-hydrolyzing conditions (blue curve) were loaded onto an SPR chip. At time zero 0.5 μM apo BtuF was injected. The experiments were repeated at least three times and error bars in (**C**) represent standard deviations of three experiments.

So far, the data show that relative to the apo state, BtuC_2_D_2_ is more stable when constantly hydrolyzing ATP, and that this stabilization is most likely due to nucleotide binding. These finding by themselves are perhaps interesting but not very surprising. What we found very unexpected is that BtuC_2_D_2_ that had worked for several hours bound more BtuF than the freshly prepared BtuC_2_D_2_ from which it originated (Fig. 3B compare black and blue curves). Importantly, pre-incubation of BtuC_2_D_2_ in any of the nucleotide-bound states did not recapitulate this ‘improvement’ effect (Fig. 3B, grey, magenta, and red curves, and see also below). Again we stress that the activity measurements were conducted after removal of nucleotides (Supplementary Fig. 6). This phenomenon was highly robust and always repeated itself, and was observed for the interaction of BtuC_2_D_2_ with both holo and apo BtuF (Fig. 4A,B). The degree of the ‘improvement’ varied between 35–60%, depending on the preparation. The data presented in Fig.4 show ~40% improvement of the worked sample (P = 7.5 × 10^−6^, n = 5, using a single preparation), with no significant changes in the equilibrium or pre-equilibrium constants (*K*_*D*_, *k*_*on*_, *k*_*off*_) of the interaction (Table 1). The only parameter that significantly differed between the naïve protein and BtuC_2_D_2_ that had worked was Rmax, representing the maximal active binding sites (BtuC_2_D_2_ molecules that are able to bind ButF). In these experiments identical amounts of freshly prepared and worked BtuC_2_D_2_ were present on the adjacent flow-cells (Supplementary Fig. 7). Since only the maximal binding changed, and not the kinetic constants, this means that relative to freshly prepared protein, a greater percentage of the BtuC_2_D_2_ molecules that had worked are in a conformation that binds BtuF (*i.e.*, have higher specific activity with respect to BtuF-binding).

Docking of BtuF to BtuC_2_D_2_ is essential for transport and this step immediately precedes translocation of vitamin B_12_. On the one hand, BtuC_2_D_2_ that had worked showed unchanged specific ATPase activity (Fig. 1D). On the other, it displayed greater specific activity with respect to BtuF-binding (Fig. 4A,B). We therefore tested whether this improved BtuF-binding activity might also be translated into accelerated transport rates. To test this we incubated BtuC_2_D_2_ for 7 hours at 37 °C under the ATP-bound or ATP-hydrolyzing conditions, and then used a rapid reconstitution protocol (see methods for details) to reconstitute pre-treated or naïve BtuC_2_D_2_ into liposomes. We verified that an equal amount of BtuC_2_D_2_ was incorporated into the liposomes (Supplementary Fig. 5C–E, Supplementary Fig. 8), and that the preparations had similar proportions of right-side-out or inside-out facing BtuC_2_D_2_ (Supplementary Fig. 8). We then measured^57^ Co-labeled vitamin B_12_ uptake into liposomes as described for Fig.2. As shown (Fig. 4C), in agreement with its improved BtuF-binding capacity, the vitamin B_12_ uptake rate of BtuC_2_D_2_ that had hydrolyzed ATP for 7 hours was ~60% faster than its fresh precursor. Importantly, incubation of BtuC_2_D_2_ in the ATP-bound state did not change its rate of vitamin B_12_ uptake (Fig. 4C), in line with its unchanged BtuF-binding activity (Fig. 3B). Collectively, these experiments show that although binding of ATP acts to stabilize BtuC_2_D_2_ and slow down its deterioration (Fig. 3A), it does not promote an increase in activity levels (relative to the fresh protein). In comparison, ATP hydrolysis by BtuC_2_D_2_ promotes stability (probably due to the dwell time in the ATP-bound state), but also leads to increased activity. The notion that only ATP-hydrolysis, and not ATP binding, leads to a more active state is also supported by experiments performed with the E159Q mutant of BtuC_2_D_2_. This mutant binds ATP but cannot hydrolyze it, yet interacts normally with BtuF^16^. However, when we incubated this mutant under the working regime we did not observe an increase in its BtuF-binding activity (Fig. 4D). This shows that the working conditions themselves, without catalysis, cannot explain enhanced activity. With wild type BtuC_2_D_2_ we very reproducibly observed that ATP binding has a stronger stabilizing effect than the working conditions (Fig. 3A, compare ATP-bound and worked after 48 hours). In contrast, when we tested the stability of the E159Q mutant in the different nucleotide-bound states we found that unlike the wild-type protein this mutant is equally stable under the working or ATP-bound conditions (Compare 48 hours of wild type in Fig. 3A to 48 hours of the mutant in Supplementary Fig. 9). Taken together these results further establish that the ATP-bound state is the most stable one, but only ATP hydrolysis leads to the hyperactive state.

### BtuC_2_D_2_ displays a conformational memory

**Figure 5:**
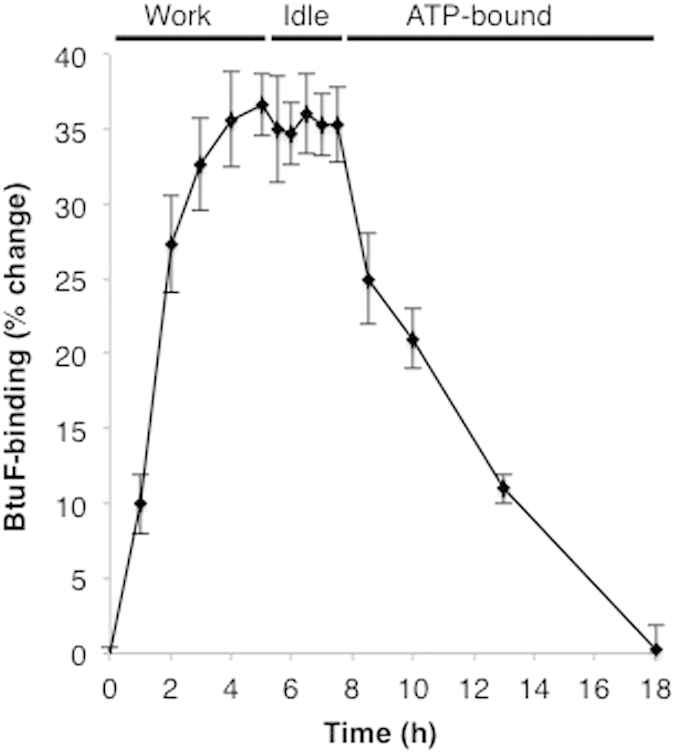
Long-term memory of BtuC_2_D_2_. Freshly prepared BtuC_2_D_2_. (time zero) was incubated under the working conditions at 37 °C (in the absence of BtuF). At the indicated intervals samples were withdrawn, ATP was removed by microfiltration, and BtuF-binding was measured by SPR. After 5 hours ATP was removed from all of the remaining volume and the protein was incubated under the idle conditions (at 4 °C, to avoid idleness-associated detrimental effects) for another 2.5 hours, during which samples were removed and BtuF-binding was measured by SPR. Subsequently, AMP-PNP was added and BtuC_2_D_2_ was moved back to 37 °C (in the ATP-bound state the protein does not deteriorate at 37 °C). At the indicated intervals samples were withdrawn, the AMP-PNP was removed by microfiltration, and BtuF-binding was measured by SPR. Results are presented as percent change from the activity of the fresh, naïve protein. Error bars represent standard deviations of triplicates from two different experiments (n = 6).

Importantly, the activity measurements shown in Figs 3 and 4 were conducted after termination of the working conditions (*i.e.,* removal of ATP-Mg). This means that the transporter remains in its excited state after the stimulus for its attainment has stopped. This phenomenon, termed “conformational memory” is not commonly observed but has been reported for horseradish peroxidase, MHC Class II molecules, cholesterol oxidase, a hairpin ribozyme, and antibody D2.3^34–38^. Common to all these examples is that ligand binding drives the conformational memory. The lengths of the conformational memories is directly related to the relaxation time of the protein from its excited state to its basal state. Depending on the protein, the conformational memory can last from milliseconds^37^ to hours^36^. To our knowledge, BtuC_2_D_2_ presents the first example for a conformational memory that is achieved by repetitive completion of a catalytic cycle. We next investigated the timescale needed to produce conformational memory in BtuC_2_D_2_, and for how long the protein remains in its ‘excited’ mode after termination of ATP hydrolysis. Short incubations (seconds, minutes) in the working conditions did not produce any detectable changes in activity levels (Supplementary Fig. 10). Rather, we found that the conditioning takes several hours, but once attained, the protein remains in its excited state for at least two hours after termination of the working conditions (Fig. 5). Notably, BtuC_2_D_2_ that reached its excited state (following ATP hydrolysis) can be shifted back to its ground state by incubation in one of the conditions that does not promote the excited state (*i.e.*, the ATP-bound state, 8–18 hours in Fig. 5). Such a behavior is consistent with a population shift phenomenon, in which the protein resides in several folded conformations. These conformations are similar, yet not identical to one another, and have different activity levels. Ligand binding (as in previously described cases^39^,^40^), or catalysis (unique for BtuC_2_D_2_) shifts the population towards one of these pre-existing conformations.

### Prolonged ATP hydrolysis induces a slow conformational change (hysteresis)

**Figure 6:**
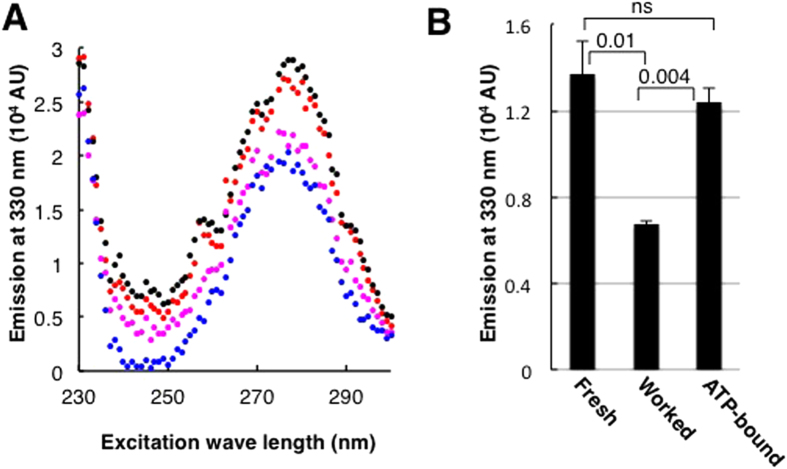
A slow conformational change of BtuC_2_D_2_ measured by quenching of tryptophan fluorescence. (**A**) BtuC_2_D_2_ was incubated for 0 (black), 1 (red), 3 (magenta) or 7 hours (blue) under the working conditions. ATP was then removed by desalting, and all samples were diluted to the same concentration. 50 μL of a 1 μM protein solution were excited at 230–300 nm, emission was measured at 330 nm. (**B**) BtuC_2_D_2_ was incubated for 16 hours under the working or the ATP-bound conditions, as indicated. Fresh protein is shown for comparison as well. ATP was removed by desalting and 50 μL of 1 μM were excited at 275 nm (peak absorbance for BtuC_2_D_2_), emission was measured at 330 nm. The experiments were repeated at least three times and error bars represent standard deviations of technical triplicates of a single experiment. Also shown are P-values obtained by a Student’s t-test (ns, not significant).

When constantly hydrolyzing ATP BtuC_2_D_2_ gradually becomes more active in terms of binding BtuF and transporting vitamin B_12_, taking several hours to reach its highest activity level. Such time-dependent changes in activity levels are termed hysteresis, and explain the cooperative behavior of monomeric proteins and other enzymes that do not conform to Michaelis-Menten principles^41^,^42^,^43^,^44^,^45^,^46^. This phenomenon originates from extremely slow conformational changes between (at least) two conformers that differ in their activity levels and/or kinetic properties^47^. Introduction of ligand, or changes in ligand concentrations, shift the population towards the more active conformer(s)^36^,^41^,^48^. When the isomerization between the conformers is slower than the enzymatic turnover rate, hysteresis is observed^47^,^49^. To monitor possible conformational changes that occur over a time scale of hours, we let BtuC_2_D_2_ hydrolyze ATP for several hours, withdrew samples, removed the nucleotides used for the pre-conditioning, and measured the protein’s tryptophan fluorescence. Figure 6A shows that during prolonged ATP hydrolysis BtuC_2_D_2_ undergoes a slow and gradual process of quenching of its 330 nm emission. Scanning the emission spectrum of the samples verified that this decrease in emission was not due to general red shift of the spectrum. Isomerization on a time scale of hours is perhaps not commonplace, but has been observed for MHC Class II molecules and for Ribozyme molecules^36^,^38^. Importantly, incubation of BtuC_2_D_2_ in the ATP-bound state did not lead to a similar quench, and its 330 nm emission was more similar to that of the naïve protein (Fig. 6B).

## Discussion

Intuitively, one might predict that a constantly working enzyme will deteriorate faster than an idle one. This is clearly not the case for BtuC_2_D_2_. The dwell time of the ATP hydrolyzing protein in the nucleotide-bound states likely accounts for this stabilization. Nevertheless, we still find this surprising: relative to the resting transporter or one that is trapped in a single nucleotide-bound state, working BtuC_2_D_2_ is much more exposed to destabilization. Unlike any single nucleotide-bound (trapped) state, it is not in equilibrium, and there is a constant flux of energy (ATP hydrolysis) that generates heat. The working protein is constantly dynamic, and likely samples a broader conformational space than the idle one. The higher dynamics present potential aggregation pitfalls and may lead to unstable conformations. Despite all these challenges, the working protein remains stable and active, much more so than the idle one. Although clearly advantageous from an evolutionary/biological perspective we find this surprising with respect to the material properties of a protein polymer.

Nucleotide binding has a strong stabilizing affect on BtuC_2_D_2_ (Fig. 3A,B), but it is insufficient to drive the hysteretic conformational change (Fig. 6B) and nor does it lead to the hyperactive state (Figs 3B, 4C,D, 5). BtuC_2_D_2_ provides the first example of hysteresis and conformational memory that are driven by catalysis, rather than by binding of a ligand to a specific conformation.

**Figure 7:**
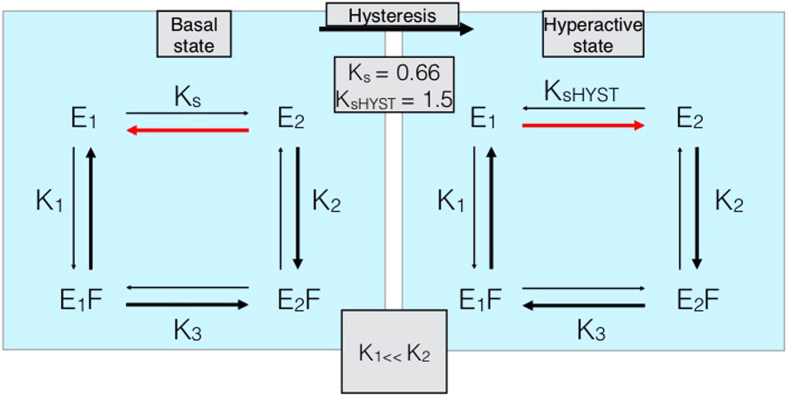
Kinetic scheme describing the hysteretic behavior of BtuC_2_D_2_. The model is written in terms of BtuC_2_D_2_ that cannot/hardly binds to BtuF (E_1_) and BtuC_2_D_2_ that binds BtuF with high affinity (E_2_). Arrows are bolded to reflect the relative reaction rates, and the red arrows depict the population shift that is affected by hysteresis. (K_1_) and (K_2_) are effective equilibrium association constants for binding of BtuF (represented as F) to BtuC_2_D_2_. K_S_, K_3_, and K_sHYST_ are effective equilibrium constants for the conformational change between E_1_ and E_2_, and are agnostic to whether the change proceeds in one step or through many intermediates. For simplicity we assume that E_1_ is transport-incompetent and that E_2_ is fully functional. The transport experiment shown in Fig.4C shows that “worked” BtuC_2_D_2_ is approximately 50% more active than the fresh protein. Therefore, since only E_2_ is transport-competent we suggest that in the initial “fresh” state of BtuC_2_D_2_ the ratio of E_2_–E_1_ is approximately 2:3 (K_s_ = 0.66). In the worked state, following the hysteretic conformational change, the E_2_/E_1_ ratio a is shifted to 3:2, *i.e.*, K_s__HYST_  =  1.5.

The hysteretic behavior of BtuC_2_D_2_ is also unique in another aspect: it presents the first example where conditioning of one function (here ATP hydrolysis) resulted in changes of activity levels of another (BtuF binding and vitamin B_12_ transport). This is especially surprising since the ATPase activity itself does not change with the hysteretic conformational change. That conditioning of one domain in the protein affects another that is 50–60 Å away is likely due to the long-range allosteric interactions that occur in BtuC_2_D_2_^50^,^51^,^52^,^53^. The opening and closing of the nucleotide binding domains (upon ATP hydrolysis) is transmitted via the coupling helices, through the transmembrane domains, to the periplasmic loops, and finally to BtuF. Another noteworthy feature of BtuC_2_D_2_ is that the hysteretic ‘excited’ state is not a single conformation but rather an ensemble of conformations that carry out the complete catalytic cycle of vitamin B_12_ transport. A highly simplified kinetic scheme describing the hysteretic behavior of BtuC_2_D_2_ is shown in Fig.7. For simplicity, shown are only two conformations (E1 and E2): E1 binds BtuF with very low affinity and therefore hardly transports vitamin B_12_; E2 is the form that interacts with BtuF with high affinity and is thus transport-competent. However, E1 and E2 each represent an ensemble of conformations that are able to complete the cycle of ATP hydrolysis, yet interact differently with BtuF. The left side of Fig.7 represents the ensemble of basal states, while the right represents the ensemble of hyperactive states. The latter are accessible to the transporter only via the hysteretic conformational change.

We have observed that continuous ATP hydrolysis increases BtuC_2_D_2_’s BtuF-binding activity. This improvement could proceed through two distinct mechanisms. First, ATP hydrolysis could gradually increase the BtuF-binding affinity of all BtuC_2_D_2_ molecules in the sample. In this case, SPR measurements would reveal an increase in BtuF binding affinity with no change of Rmax for the worked sample over the fresh sample. Alternatively, ATP hydrolysis could drive a shift in the relative ratio of coexisting BtuF-incompetent (E1) and BtuF-competent (E2) conformations of BtuC_2_D_2_ (Fig. 7). In this case, SPR measurements comparing worked and fresh BtuC_2_D_2_ samples would detect no change in BtuF binding affinity while Rmax would increase. As seen in Table 1, the second, population shift mechanism is consistent with experimental observation. The maximum number of binding events (Rmax), increases from 67.7 in the fresh sample to 93.02 in the worked sample, a ~40% increase. From this data, we deduce that the population of BtuC_2_D_2_ molecules capable of binding BtuF increased by 40%. This is consistent in a shift in the ratio E2/E1 from 0.66 (2/3) in the fresh sample (Fig. 7, left) to 1.5 (3/2) in the worked sample (Fig. 7, right). The hysteretic conformational change is very slow, and occurs over several hours (Fig. 6). Likewise, attainment of the hyperactive state also proceeds over several hours (Fig. 5). In our view these two processes are linked and are practically synonymous. Since all other catalytic steps occur on a time-scale of seconds we believe that the rate-limiting step is the conversion between the E1 and E2 conformations.

How common is hysteresis and conformational memory in proteins?

In addition to BtuC_2_D_2_ we tested two other ABC transporters (MetIN and ModBC) and found no evidence for hysteresis or conformational memory. It therefore seems that it is not a common feature of ABC transporters. We thoroughly surveyed the literature and found a handful of reported examples of enzymes/proteins that display conformational memory^34^,^35^,^36^,^37^,^38^,^39^. However, we believe that this may be an underrepresentation that stems from technical limitations: when the isomerization is very slow and the conformational memory is long, bulk measurements can identify hysteretic behavior (herein and in MHC Class II molecules^36^). However, faster isomerization that leads to a conformational memory that lasts seconds or milliseconds will often require single molecule measurements that are available for very few systems.

The *in-vivo* relevance of our observation with BtuC_2_D_2_ remains unknown: we do not know whether in the cell BtuC_2_D_2_ attains a hyperactive state following prolonged activity. However, it is tempting to tie our observations to a long lasting yet puzzling observation that BtuC_2_D_2_ has a very high basal ATPase activity in the absence of its transported substrate^16^,^17^. In light of the results reported here this constant activity may maintain its structural and functional integrity and keep it in a more active state. Considering the limited capacity of membranes to contain proteins, the existence of such an inherent mechanism that stabilizes and improves the activity of membrane-embedded enzymes only for as long as they are active (and needed) is clearly beneficial.

## Materials and Methods

### Protein expression and purification

His-tagged BtuC_2_D_2_ and FLAG-tagged BtuF were over-expressed and purified as was extensively described before^17^,^54^. Briefly, BtuC_2_D_2_ was overexpressed from single plasmid containing an amino terminal histidine tag on BtuC. Cells lysate was solubilized in 1% dodecyl-*N*,*N*-dimethylamineoxide (LDAO, Affymetrix) and BtuC_2_D_2_ was purified using Ni-NTA metal affinity chromatography. FLAG-tagged BtuF was purified from osmotic shock extracts by size exclusion chromatography.

### Incubation regimes

50–100 μg BtuC_2_D_2_ (1 mg/mL) were dialyzed in the dark at the indicated temperatures against a 1000-fold excess of buffer containing 25 mM Tris·HCl, pH 7.5, 150 mM NaCl, 50 μM EDTA, 0.08% (w/v) N-dimethylamine-N-oxide (LDAO, Affymetrix), 0.008 mg/mL *E. coli* lipids. LDAO and *E.coli* lipids were omitted from experiments done in nanodiscs 100 μM ATP and 1mM MgCl_2_ were added to set the working regime, and ATP was omitted for the resting condition. To ensure a fresh supply of ATP the buffer was changed every 12-20 hours. The following conditions were used to mimic the intermediate bound states: ATP-bound: 100 μM Adenosine 5′-(β,γ-imido) triphosphate (AMP-PNP, Sigma)/ 1mM MgCl_2_ or 100 μM ATP/50 μM EDTA. Others and we have previously observed that both conditions mimic the ATP bound state^28^,^31^,^50^. ADP bound: 100 μM ADP/1mM MgCl_2._ The transition state was trapped as previously described^28^ using 300 μM ortho-vanadate in the presence of 100 μM ADP/ 1mM MgCl_2_. Samples were withdrawn at the indicated time points and centrifuged before analysis (10 minutes 17,000 x g, or 30 minutes 150,000 x g as indicated).

### ATP hydrolysis assays

The hydrolysis of ATP, GTP, or CTP was measured using Molecular Probes (USA) EnzCheck® kit, at 28 °C in a 96-well format, according to the manufacturer’s specifications and as described in^55^. Each well contained 100 μL of 0.1–1 μM BtuC_2_D_2_ (as indicated) in 25 mM Tris·HCl, pH 7.5, 150 mM NaCl, 50 μM EDTA, 0.008 mg/mL *E. coli* lipids, and 0.08% (w/v) N-dimethylamine-N-oxide (LDAO, Affymetrix). LDAO was omitted from experiments with reconstituted nanodiscs and liposomes. To initiate hydrolysis, MgCl_2_ was injected to a final concentration of 2 mM (or 1mM in assays with liposomes).

### SPR measurements

The procedure for measuring the interaction between BtuC_2_D_2_ and BtuF using SPR had been extensively described^28^,^29^. Briefly, BtuC_2_D_2_ (15–20 ng, 1500–2000 RU) was immobilized onto a Ni-NTA biosensor chip (GE healthcare) and FLAG-tagged BtuF was injected at the indicated concentration. All measurements were conducted on using a Biacore™ T200 (GE healthcare), at 25 °C, and the block compartment was cooled to 7 °C. Experiments were run in 50 mM Tris-HCl pH 7.5, 150 mM NaCl, 0.1% (w/w) n-dodecyl-N, N-dimethylamine-N-oxide (LDAO) at 15 μL min^−1^ (mass transport limitations were not observed). Where appropriate, ligands (vitamin B_12_, nucleotides) were added only to the analyte’s buffer (BtuF). Double referencing was applied to all sensograms.

### Reconstitution of BtuC_2_D_2_

BtuC_2_D_2_ was reconstituted essentially as previously described^17^ with minor modifications. Egg phosphatidylcholine and *E. coli* polar lipids (Avanti) were mixed in chloroform in a 1:3 ratio (w/w). Chloroform was removed under a gentle nitrogen stream and residual chloroform was removed by over-night vacuum. The lipids were re-suspended in nitrogen-flushed 20 mM Tris-HCl pH 7.5 at 10 mg/mL by vortexing and sonication in a bath sonicator. Lipids (10 mg/mL) were stored at −80 °C until use. For reconstitution, lipids were thawed in a room temperature water bath, subjected to three rounds of freezing (liquid nitrogen) and thawing (water bath), and extruded 11 times through a double layer of 400 nm polycarbonate filters (using Avanti Polar Lipids Mini-Extruder). Purified BtuC_2_D_2_ (1mg/mL in 0.1% LDAO) was then added at a 1:50 ratio (w/w) of protein/lipids and the mixture was gently agitated for 30 minutes at room temperature. BioBeads SM2 (BioRad) were used to remove the detergent. Before their use, the BioBeads were activated by washing (once) with methanol, three times with ethanol, and five times with water. BioBeads were added at 40 mg/mL for 15 minutes at room temperature, 15 minutes at 4 °C, 30 min at 4 °C, overnight at 4 °C, and finally 1 hour at 4 °C. Fresh beads were used for in each exchange. The proteoliposomes were harvested by ultracentrifugation (20 minutes, 150,000 x g), washed with nitrogen-flushed 20 mM Tris-HCl pH 7.5, re-suspended in the same buffer to 10 mg/mL, frozen in liquid nitrogen and stored in −80 °C.

### Rapid reconstitution of BtuC_2_D_2_

The protocol used for reconstituting primed BtuC_2_D_2_ (shown in Fig. 4C) is similar to the one described above with the some modifications. Prior to reconstitution of pre-conditioned BtuC_2_D_2_ the liposomes were loaded with an ATP regenerating system (see below). Subsequently, all steps were carried in 4 °C, and following a 20-minute incubation of primed BtuC_2_D_2_ with the pre-formed, pre-loaded liposomes the detergent was immediately removed by 4 successive incubations with 160 mg/mL SM-2 bio beads, each incubation for 15 minutes. The proteoliposomes were then diluted 3-fold with detergent free buffer, collected by ultracentrifugation (15 minutes, 150,000 x g) rinsed once (without re-suspension) with 1 ml of 20 mM Tris-HCl pH 7.5, 150 mM NaCl, 2 mM MgCl_2_, and re-suspended to 10 mg/mL. For transport assays with reconstituted pre-conditioned BtuC_2_D_2_ the proteoliposomes were immediately used for transport assays, without freezing.

### Sucrose floatation assays

To remove proteins that are not membrane embedded, 50 ul of proteoliposomes used in the transport assays were washed with 100 mM Na_2_CO_3_, 20 mM Tris-HCl pH 7.5, 150 mM NaCl. Proteoliposomes were then placed at the bottom of ultracentrifugation tube and density cushions of 60, 40, and 0% (w/v) sucrose were built on top (volumes of 0.95 ml, 2 ml, and 1 ml respectively). Samples were centrifuged (50,000 X g, 4 °C, 16 hours) using ultracentrifuge in swinging rotor (SW TH660). Ten fractions of 0.4 ml each were gently collected and analyzed by western blot using anti His antibody.

### Transport assays in proteoliposomes

Uptake of vitamin B_12_ was measured using previously established protocol^17^ with several modifications. To incorporate an ATP regenerating system the proteoliposomes suspension was supplemented with 1 mM Na-ATP, 2 mM MgCl_2_, 20 mM creatine phosphate, and 0.06 mg/mL creatine kinase. The proteoliposomes were then subjected to three cycles of freeze/thaw and extruded 11 times through a double layer of 400 nm polycarbonate filters. Following one wash with 20 mM Tris-HCl pH 7.5, 150 mM NaCl, 2 mM MgCl_2_, the proteoliposomes were re-suspended with the same buffer to 10 mg/mL lipids. All steps were preformed in 4 °C and the proteoliposomes were kept on ice. To initiate transport, the proteoliposomes were diluted to 5 mg/mL, heated to 33 °C for 2 minutes, and combined with 0.5 μM BtuF/5 μM^57^ Co-labeled vitamin B_12_ (MP biomed, 1.67 μCi/mL). At the indicated intervals a 50 μL sample was removed, diluted with 4 ml of ice-cold 20 mM Tris-HCl pH 7.5, 150 mM NaCl, 2 mM MgCl_2_, 100 μM unlabeled vitamin B_12_, and immediately filtered through 0.2 μM cellulose acetate filters. The filters were washed once with 4 ml of the same ice-cold buffer. The amount of ^57^ Co-labeled vitamin B_12_ on the filters was determined using a γ counter.

### Nanodisc reconstitution

The method for self-assembly of membrane protein in monodisperse phospholipid bilayer, held by a scaffold protein was established in^30^ and used for ABC transporters. Nanodiscs containing BtuC_2_D_2_ were prepared using tag-less membrane scaffold protein (MSP1-E3D1) and lipids as describe in^31^. Briefly, purified BtuC_2_D_2_ complex in LDAO was mixed with purified, TEV cleaved MSP1-E3D1 and lipids (*E. coli* polar lipids and phosphatidylcholine at a 3:1(w/w) ratio, and solubilized in 4% Triton X-100) to final concentrations of 4 μM, 24 μM and 0.85 mM respectively. After a 30-min incubation under gentle agitation at room temperature, detergents were removed by BioBeads, with 250 mg BioBeads per 1 ml mixture, 15 min at room temperature followed by 3 incubations of 15, 30, and 120 minutes at 4 °C (fresh beads were used each time).

### Tryptophan fluorescence measurements

Nucleotides were removed from the protein samples with Zeba^TM^ Spin Desalting columns, 40 K MWCO (Thermo Scientific) and the samples were diluted to 0.2 mg/mL with the dialysis buffer (excluding the nucleotides). Triplicates of 50 μL were excitation-scanned (Ex 230–440 nm, Em 330 nm) using a monochromator-based Tecan M200 plate reader.

## Additional Information

**How to cite this article**: Livnat-Levanon, N. *et al.* The uncoupled ATPase activity of the ABC transporter BtuC_2_D_2_ leads to a hysteretic conformational change, conformational memory, and improved activity. *Sci. Rep.* **6**, 21696; doi: 10.1038/srep21696 (2016).

## Supplementary Material

Supplementary Information

## Figures and Tables

**Table 1:** Kinetic rate constants (determined by SPR) for the interaction of apo BtuF with fresh BtuC_2_D_2_ or BtuC_2_D_2_ that had worked for 7 hours.

	Fresh	Worked	T. test
*k*_*on*_ (10^5^ M^−1^, s^−1^)	2.8 ± 0.7	2.9 ± 0.6	ns
*k*_*off*_ (10^−5^ s^−1^)	1.2 ± 0.3	1.1 ± 0.4	ns
*K*_*d*_ (10^−11^ M)	3.6 ± 0.8	3.7 ± 0.5	ns
Rmax (Ru)	67.7 ± 1.8	93.3 ± 1.4	9.20E-06

Rmax describes the maximal number of binding events and is given in response units (Ru). Errors represent standard deviations of five independent experiments.
