# Supplementary material for: The uncoupled ATPase activity of the ABC transporter BtuC2D2 leads to a hysteretic conformational change, conformational memory, and improved activity
Source: Sci Rep. 2016 Feb 22;6:21696. doi: 10.1038/srep21696 (PMC4765350; doi:10.1038/srep21696)
Supplement: Supplementary Information [file srep21696-s1.pdf]

Supporting information.

# The uncoupled ATPase activity of the ABC transporter BtuCD leads to a hysteretic conformational change, conformational memory, and improved activity.

Nurit Livnat-Levanon[1], Amy Gilson[2], Nir Ben-Tal[3], and Oded Lewinson[1].

[1]Department of Biochemistry, The Bruce and Ruth Rappaport Faculty of Medicine, Technion-Israel Institute of Technology, Haifa, Israel.

[2]Department of Chemistry and Chemical Biology, Harvard University, Cambridge, MA, USA.

[3]Department of Biochemistry and Molecular Biology, Tel-Aviv University, Tel-Aviv, Israel.

Corresponding author: Oded Lewinson
e-mail: lewinson@tx.technion.ac.il;
telephone: +972-4-8295428;
fax: +972-4-8295205

SUPPLEMENTARY FIGURE LEGENDS

**Supplementary Figure 1.** (A) ATP hydrolysis by freshly prepared $BtuC_2D_2$ in LDAO. The blue diamonds is the experimental data and the dashed black curve is Michaelis-Menten fit of the data. Also shown are the kinetic rate constants Vmax, Km, and the Hill coefficient. (B) ATP is not depleted over 24 hours of incubation under the 'working' regime. Initial rates of ATP hydrolysis by 1 μM $BtuC_2D_2$ of fresh 100 μM ATP (red curve) or the same buffer that was dialyzed (1000-fold excess of buffer) for 24 hours at 28° C with a micro-dialysis tube containing 6 μM $BtuC_2D_2$ (blue curve). This dialysis regime (100 μM ATP, 6 μM $BtuC_2D_2$, 28° C, 1000-fold excess of buffer, and buffer replacement every 16-24 hours) was used in all 'working' regime experiments conducted at 28° C.

**Supplementary Figure 2.** Aggregation of rested and worked $BtuC_2D_2$. (A) $BtuC_2D_2$ was incubated for at 28° C under the working (black) or resting (red) conditions. After 2 days samples were withdrawn and centrifuged for 10 minutes at 17,000 xg. 10 μg from each were injected onto an analytical gel filtration column (Superdex200 5/15, $V_0$= 1.1 mL, $V_t$= 3.3 mL). (B) $BtuC_2D_2$ was incubated for at 28° C under the working or resting conditions, as indicated. After 9 days, the solution was thoroughly pipetted out and the precipitated protein recovered from the dialysis tubes by treatment with 10% SDS. Shown is a Coomassie blue staining of a reducing SDS-PAGE of equal volumes from such SDS extractions. The fainter lower bands (~25 kDa) are the expected molecular

weight of BtuC and BtuD (the $BtuC_2D_2$ complex falls apart in SDS gels), while the higher bands (~63 kDa), are SDS-resistant higher oligomers that are mostly observed in the rested sample.

**Supplementary Figure 3.** The effect of temperature on the deterioration of the resting and working protein. ATPase activity of the worked (black bars) and rested (white bars) $BtuC_2D_2$ after 48 hours of incubation at the indicated temperatures. The experiment was repeated three times and the error bars represent the standard deviation of technical repeats from a single experiment (n=3).

**Supplementary Figure 4.** Hydrolysis of ATP, GTP, and CTP by rested or worked $BtuC_2D_2$. $BtuC_2D_2$ was incubated for 48 hours at 37°C under the resting or working regimes (as indicated) and then assayed for hydrolysis of 1mM ATP, CTP or GTP, as indicated. The experiment was repeated three times and the error bars represent the standard deviation of technical repeats from a single experiment (n=3).

**Supplementary Figure 5.** Sucrose floatation assays of $BtuC_2D_2$-liposomes. Proteoliposomes were washed with $Na_2CO_3$ (to remove proteins that are not membrane embedded) and subjected to sucrose floatation assays (as detailed in the materials and methods). 10 fractions of equal volume were analyzed by western blot using anti His antibody. Fraction 1 is the top-most fraction and 10 is the bottom most. (A) and (B) Analysis of the proteoliposomes used in the transport assays shown in Figure 2B. (C), (D), (E) Analysis of the proteoliposomes used in the transport assays shown in Figure 4C. As shown, for all of the preparations $BtuC_2D_2$ is found only in fractions 2-4 that is the interface between 0% and 40% sucrose, as expected for a membrane-embedded protein.

In none of the preparations $BtuC_2D_2$ is found in the bottom fractions (corresponding to aggregates).

**Supplementary Figure 6.** Removal of ATP by microfiltration. (A) Shown is the 260nm to 280nm absorbance ratio of 6 μM $BtuC_2D_2$, 6 μM $BtuC_2D_2$ + 100 μM ATP, and 6 μM $BtuC_2D_2$ + 100 μM ATP after desalting using spin micro filters. Error bars represent the standard deviation of three technical repeats from two independent experiments (n=6). (B) 50 μL from the same samples of A were injected onto a 24 mL Superdex 200 Increase gel filtration column ($V_0$= 8 mL, $V_t$= 24 mL). Shown is the 260 nm absorbance of 6 μM $BtuC_2D_2$ (blue), 6 μM $BtuC_2D_2$ + 100 μM ATP (red), and 6 μM $BtuC_2D_2$ + 100 μM ATP after desalting using spin micro filters (green).

**Supplementary Figure 7.** Loading of identical amounts of fresh $BtuC_2D_2$ (red) or $BtuC_2D_2$ that had worked for 7 hours (blue). Both samples were diluted approximately to 0.02 mg/mL and initially injected for 225 seconds to adjacent flow-cells of a Ni-NTA biosensor chip. Since after the first injection the fresh protein was immobilized at a somewhat higher level, a second injection was performed at 450 seconds, but only of worked $BtuC_2D_2$. This injection was stopped at 550 seconds to achieve identical immobilization of the two samples.

**Supplementary Figure 8.** Content and orientation of $BtuC_2D_2$ in the liposomes. (A) Reconstituted $BtuC_2D_2$ may insert in the right side out orientation (molecule #1) or inside side out orientation (molecule #2) (ARS, ATP Regenerating System). The transport experiments of Figure 4C measure only the activity of $BtuC_2D_2$ in orientation #1, and are 'blind' to $BtuC_2D_2$ molecules in orientation #2. The increased transport activity of worked $BtuC_2D_2$ can result from the higher protein content in these lipoosomes. Panel (B) shows

that this is not the case: Shown is a silver stain of a reducing SDS-PAGE of 10 μL each from the liposomes preparations used in the vitamin $B_{12}$ transport experiments shown in Figure 4C. As indicated, shown are empty liposomes, liposomes reconstituted with fresh $BtuC_2D_2$, with $BtuC_2D_2$ that was pre-incubated in the ATP-bound state for 7 hours, or with $BtuC_2D_2$ that had worked for 7 hours.

An alternative explanation to the increased transport activity of worked-$BtuC_2D_2$ liposomes is that they have (relative to naïve- $BtuC_2D_2$ in liposomes) a greater fraction of the molecules in orientation #1. This was tested indirectly by ATP hydrolysis assays (panel C, error bars represent the standard deviation of three measurements) that measure the activity of molecules in orientation #2. 1 mM ATP-Mg was added (externally) to a 100 μL suspension of liposomes reconstituted with fresh $BtuC_2D_2$ (red curve), or liposomes that were reconstituted with $BtuC_2D_2$ that had been incubated for 7 hours in the ATP-bound state (blue curve), or working conditions (green curve). As shown, the worked-$BtuC_2D_2$ liposomes had the highest rate of ATP hydrolysis. These liposomes have the same protein content as the other liposomes' preparations (panel B), and worked $BtuC_2D_2$ has the same specific ATPase activity as fresh protein (Figure 1D). This means that worked-$BtuC_2D_2$ liposomes have the highest proportion of inside out oriented molecules (orientation #2) and the lowest proportion of transport-competent oriented molecules (orientation #1). Therefore, the increased vitamin $B_{12}$ transport activity observed with worked-$BtuC_2D_2$ liposomes cannot be explained neither by a higher protein content in the liposomes, nor by preferred directionality of the membrane insertion of worked-$BtuC_2D_2$.

**Supplementary Figure 9.** Stabilization of the E159Q mutant by nucleotides. $BtuC_2D_2$ (E159Q) was incubated at the indicated conditions at 28°C for 24 or 48 hours, as indicated. The amount of protein that remained in the soluble fraction visualized by coomassie staining of reducing SDS-PAGE. The gels were identically run, stained, and imaged.

**Supplementary Figure 10.** Short periods of ATP hydrolysis do not improve BtuF binding. Equal amounts of $BtuC_2D_2$ were immobilized onto adjacent flow cells on a biosensor chip and washed for 5 minutes in the absence (blue) or presence (red) of 100 μM ATP/1mM $Mg^{2+}$. Apo BtuF (0.25 μM) was then immediately injected and the association was recorded by SPR.

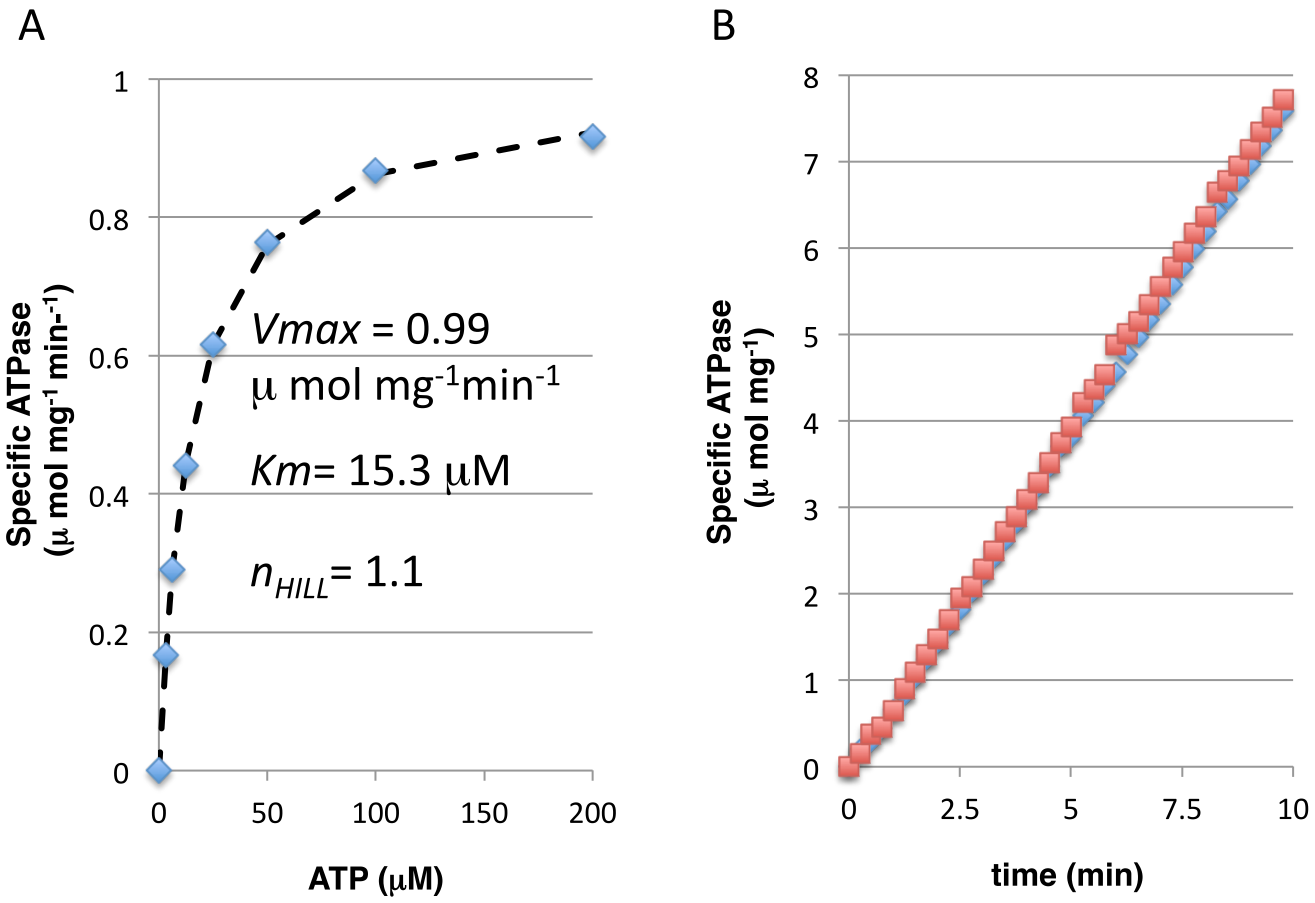
A
B
1
0.8
0.6
0.4
0.2
0
0
50
100
150
200
ATP (μM)
Specific ATPase (μ mol mg-1 min-1)
Vmax = 0.99 μ mol mg-1min-1
Km= 15.3 μM
nHILL= 1.1
8
7
6
5
4
3
2
1
0
0
2.5
5
7.5
10
time (min)
Specific ATPase (μ mol mg-1)

Figure S1

A B

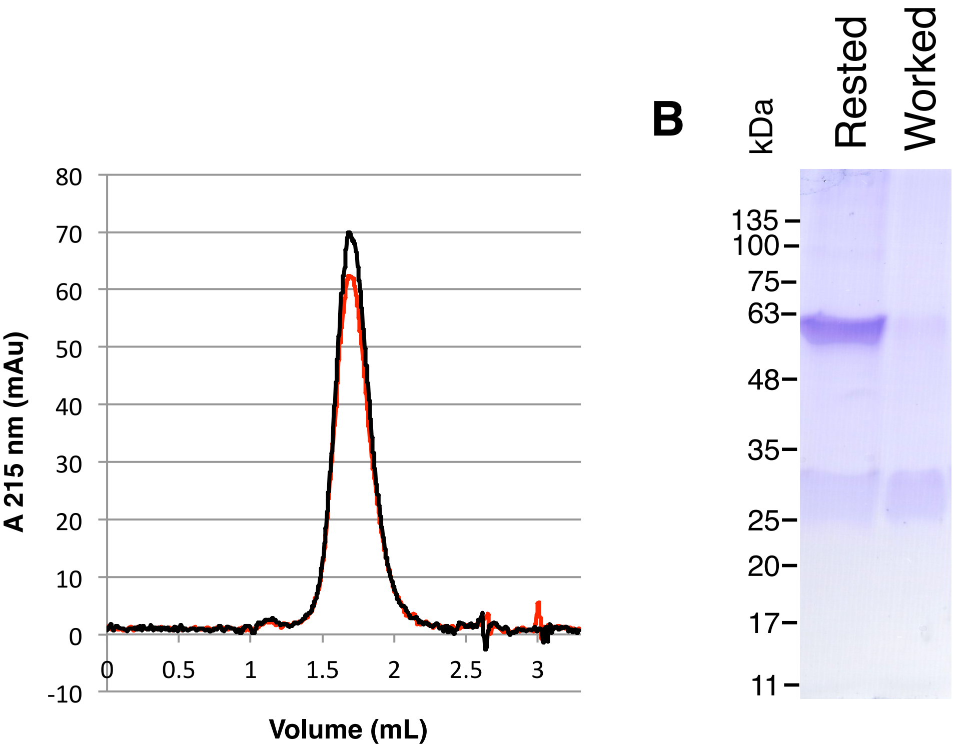
80
70
60
50
40
30
20
10
0
-10
A 215 nm (mAu)
0
0.5
1
1.5
2
2.5
3
Volume (mL)
kDa
Rested
Worked
135
100
75
63
48
35
25
20
17
11

Figure S2

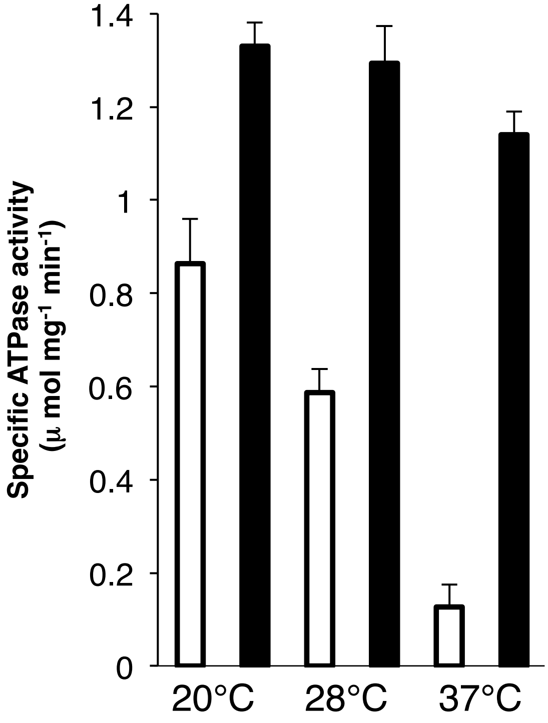
Specific ATPase activity
(μ mol mg-1 min-1)
1.4
1.2
1
0.8
0.6
0.4
0.2
0
20°C
28°C
37°C

Figure S3

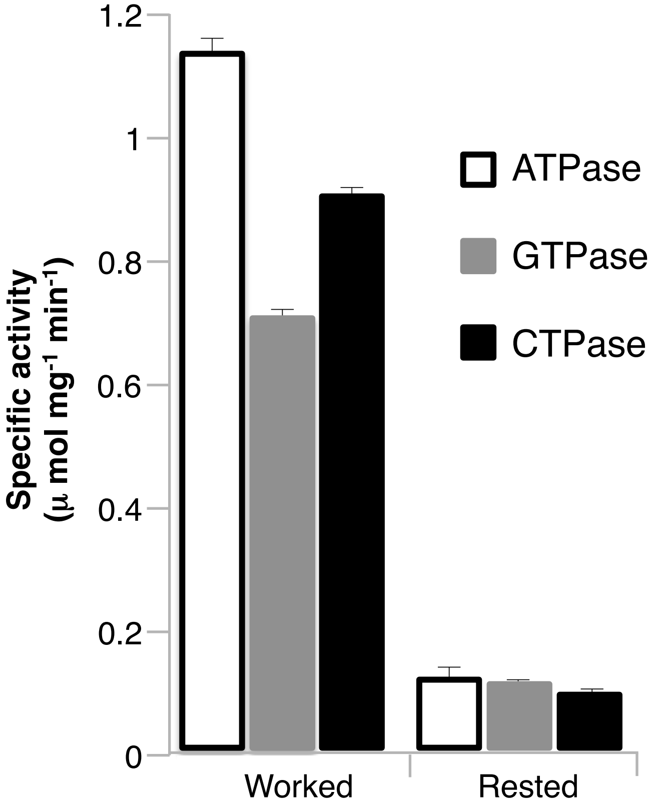
1.2
1
0.8
0.6
0.4
0.2
0
Specific activity
(μ mol mg-1 min-1)
ATPase
GTPase
CTPase
Worked
Rested

Figure S4.

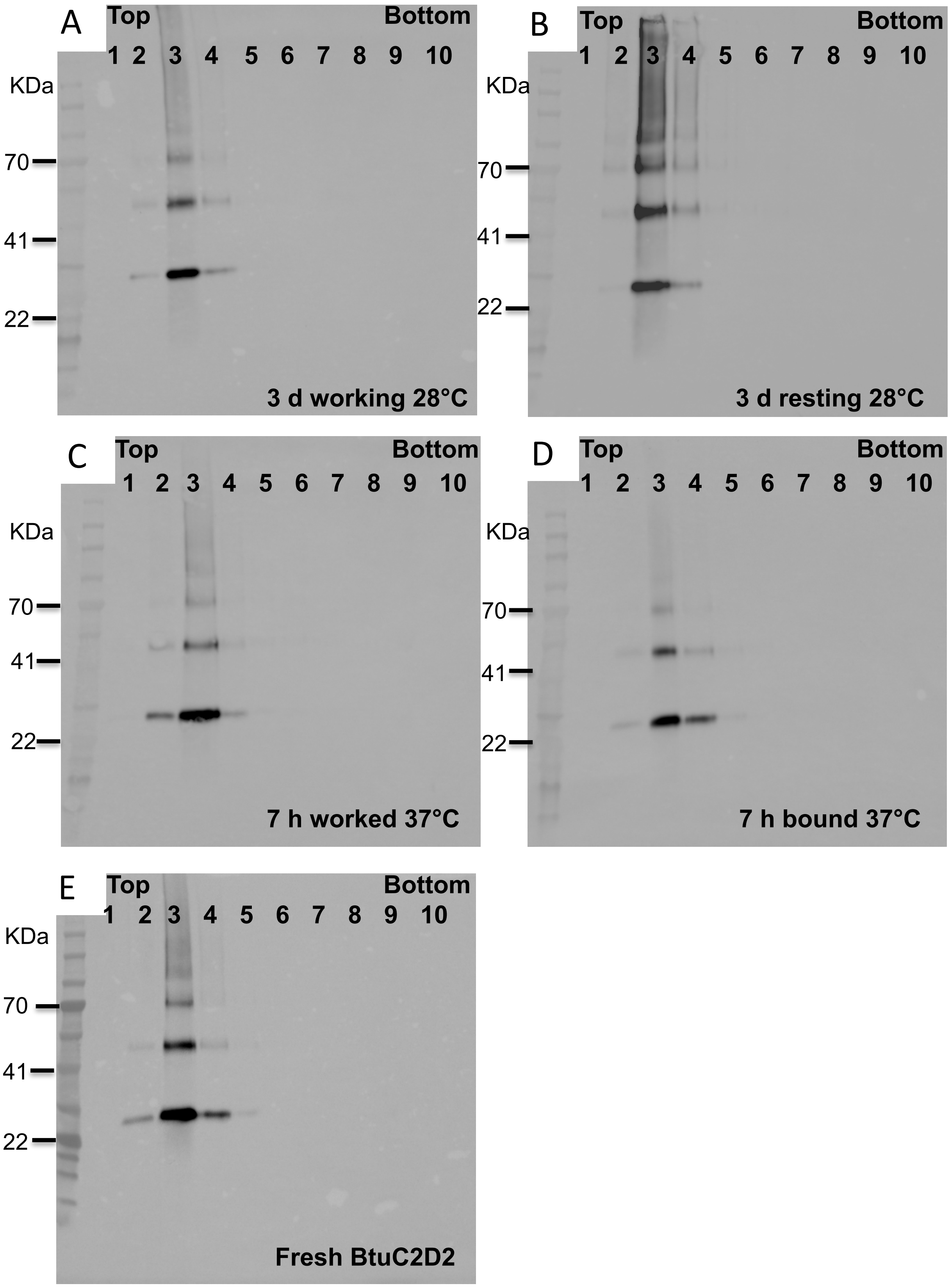
A
Top
Bottom
1 2 3 4 5 6 7 8 9 10
KDa
70
41
22
3 d working 28°C
B
Top
Bottom
1 2 3 4 5 6 7 8 9 10
KDa
70
41
22
3 d resting 28°C
C
Top
Bottom
1 2 3 4 5 6 7 8 9 10
KDa
70
41
22
7 h worked 37°C
D
Top
Bottom
1 2 3 4 5 6 7 8 9 10
KDa
70
41
22
7 h bound 37°C
E
Top
Bottom
1 2 3 4 5 6 7 8 9 10
KDa
70
41
22
Fresh BtuC2D2

Figure S5.

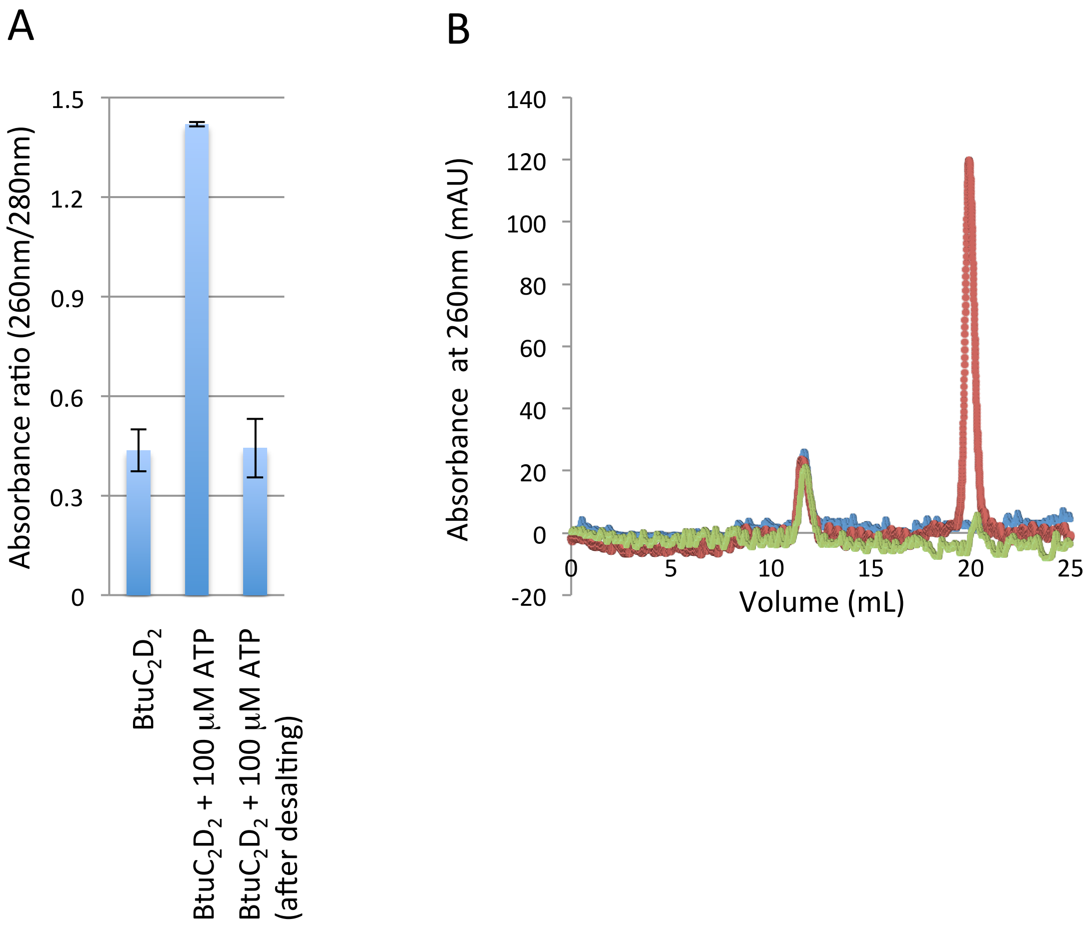
A
B
Absorbance ratio (260nm/280nm)
1.5
1.2
0.9
0.6
0.3
0
BtuC2D2
BtuC2D2 + 100 μM ATP
BtuC2D2 + 100 μM ATP (after desalting)
Absorbance at 260nm (mAU)
140
120
100
80
60
40
20
0
-20
0
5
10
15
20
25
Volume (mL)

Figure S6.

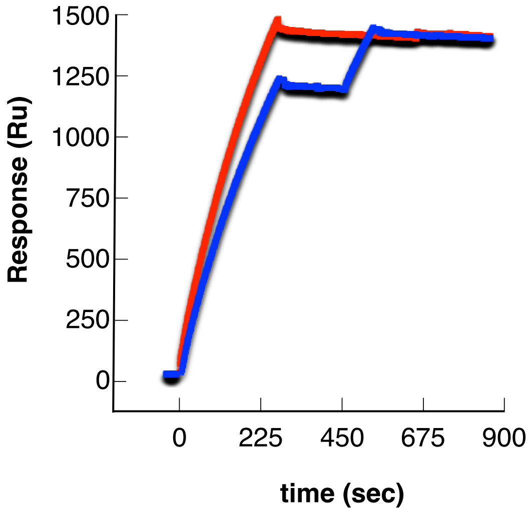

1500
1250
1000
750
500
250
0
Response (Ru)
0
225
450
675
900
time (sec)


Figure S7.

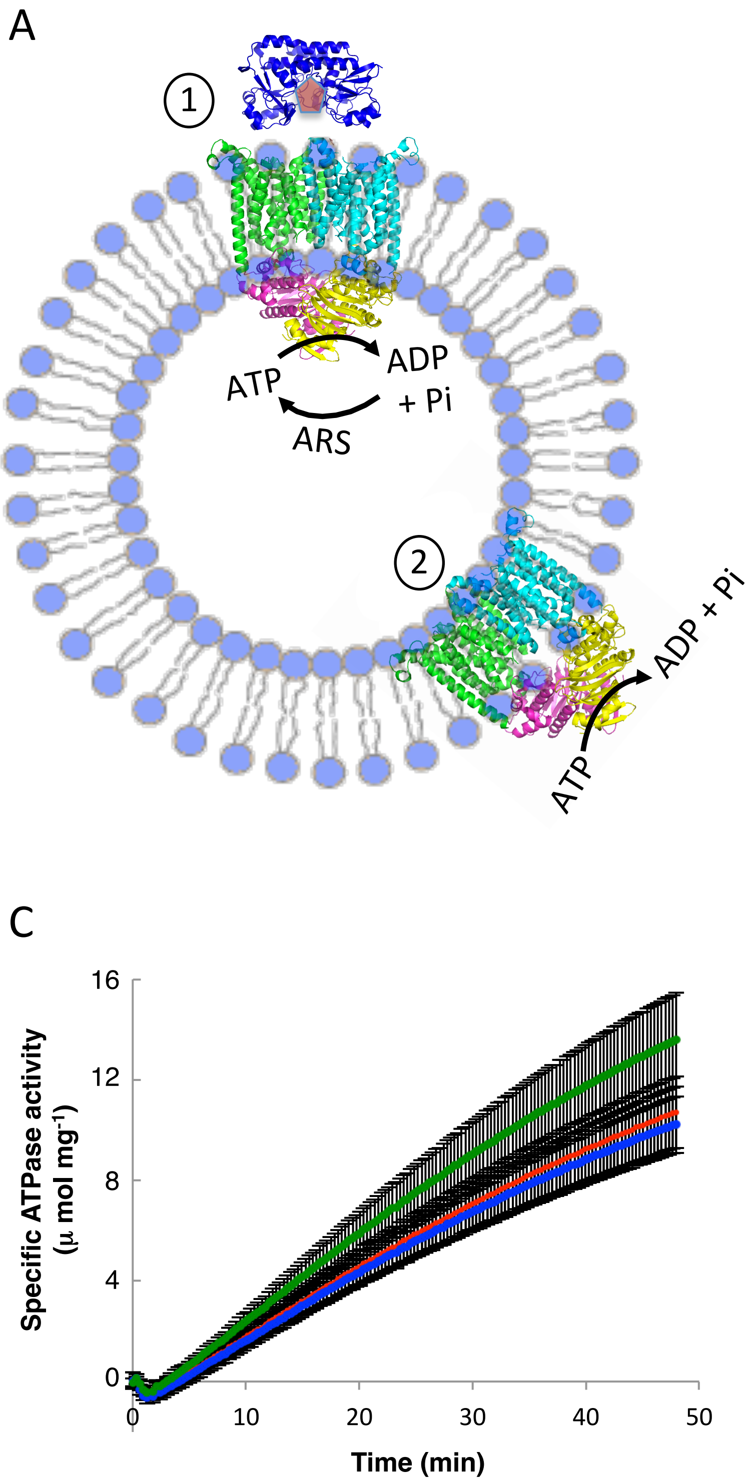
A
1
ATP
ADP
+ Pi
ARS
2
ATP
ADP + Pi
B
kDa
Empty
Fresh
ATP bound
Worked
C
16
12
8
4
0
0
10
20
30
40
50
Specific ATPase activity
(μ mol mg-1)
Time (min)

Figure S8.

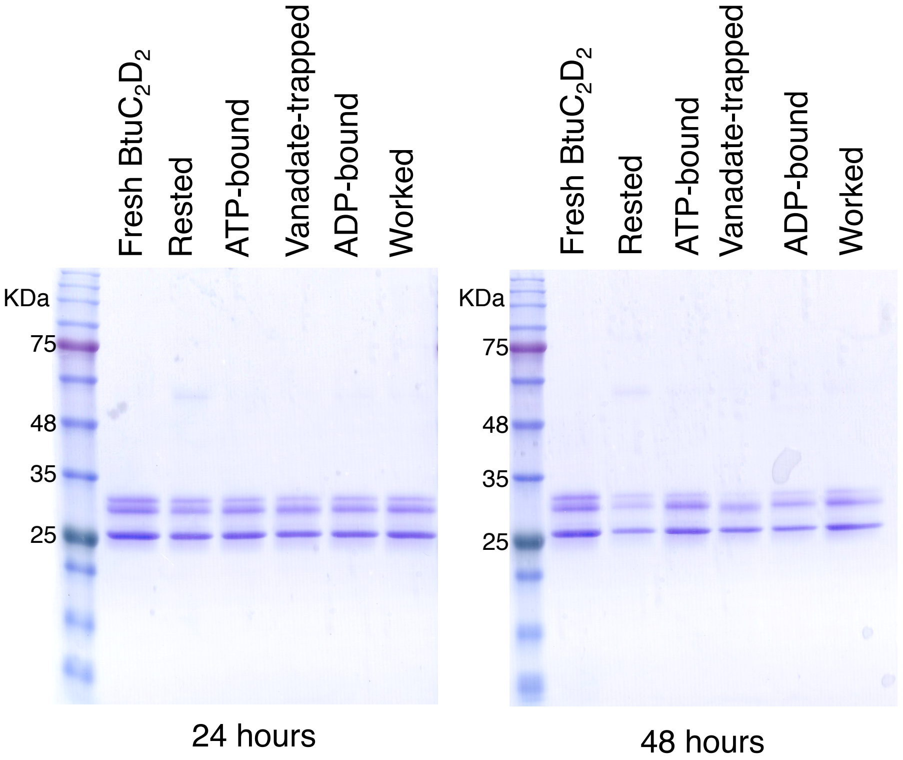
Fresh $BtuC_2D_2$
Rested
ATP-bound
Vanadate-trapped
ADP-bound
Worked
KDa
75
48
35
25
24 hours
Fresh $BtuC_2D_2$
Rested
ATP-bound
Vanadate-trapped
ADP-bound
Worked
KDa
75
48
35
25
48 hours

Figure S9.

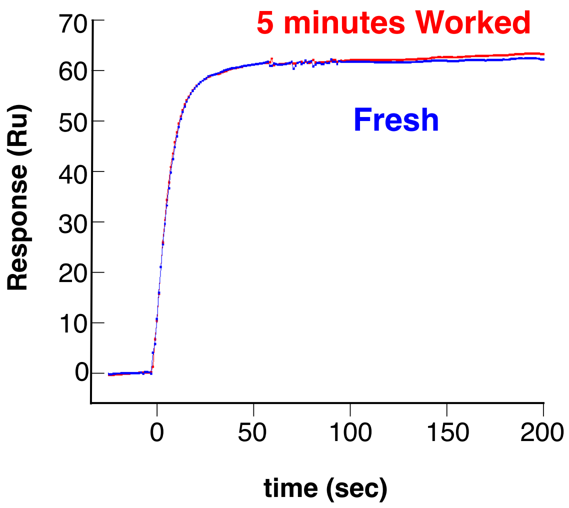
5 minutes Worked
Fresh
Response (Ru)
70
60
50
40
30
20
10
0
0
50
100
150
200
time (sec)

Figure S10.
